# Efficacy and safety of immune checkpoint inhibitor consolidation after chemoradiation in patients of Asian ethnicity with unresectable stage III non‐small cell lung cancer: Chinese multicenter report and literature review

**DOI:** 10.1111/1759-7714.13631

**Published:** 2020-08-24

**Authors:** Tao Zhang, Kunpeng Xu, Nan Bi, Li Zhang, Wei Jiang, Jun Liang, Lei Deng, Xin Wang, Jianyang Wang, Jie Wang, Luhua Wang

**Affiliations:** ^1^ Department of Radiation Oncology, National Cancer Center/National Clinical Research Center for Cancer/Cancer Hospital Chinese Academy of Medical Sciences and Peking Union Medical College Beijing China; ^2^ Department of Oncology, Tongji Hospital, Tongji Medical College Huazhong University of Science and Technology Wuhan China; ^3^ Department of Radiation Oncology, National Cancer Center/Cancer Hospital & Shenzhen Hospital Chinese Academy of Medical Sciences and Peking Union Medical College Shenzhen China; ^4^ Department of Medical Oncology, National Cancer Center/National Clinical Research Center for Cancer/Cancer Hospital Chinese Academy of Medical Sciences and Peking Union Medical College Beijing China

**Keywords:** Chinese, PD‐1, PD‐L1, pneumonitis

## Abstract

**Background:**

The PACIFIC study has defined a new standard of care for patients with unresectable stage III non‐small cell lung cancer (NSCLC) in the form of immune checkpoint inhibitor (ICI) consolidation therapy. However, there is little specific data pertaining to the safety and efficacy of this approach in Chinese NSCLC patients.

**Methods:**

This was a prospective multicenter cohort study. Between September 2018 and January 2020, patients with unresectable stage III NSCLC that had undergone chemoradiation therapy (CRT) and ICI consolidation treatment were enrolled in this study. The short‐term safety, tolerability, and efficacy of ICI combination with CRT were evaluated in these patients.

**Results:**

Of the 20 Chinese patients eligible for inclusion, 17 (85.0%) underwent concurrent CRT treatment. In these patients, a median period of 40.5 days (range: 1–85 days) passed between the end of CRT and initiation of consolidation therapy. Pneumonitis occurred in 80.0% of patients, with seven (35.0%) being diagnosed with grade 1 pneumonitis and nine (45.0%) with grade 2 pneumonitis. No patients experienced grade 3 or higher pneumonitis or other ICI‐related toxicities. Lung V20 ≥ 20% was associated with higher grade 2 pneumonitis (77.8%; ≥20% vs. 18.2%; <20%, *P* = 0.027). The overall response rate (ORR) in these patients was 95.0%. Over a median follow‐up period of 11.3 months (range: 6.2–21.8 months), 12‐month PFS of these patients were 89.5% (95% CI: 76.7–100.0%), and 12 months OS was 100.0%.

**Conclusions:**

These data indicate that ICI consolidation therapy can achieve favorable short‐term efficacy, while exhibiting good safety and acceptable toxicity profiles in Chinese patients with unresectable stage III NSCLC.

**Key points:**

**Significant findings of the study**

This is the first report evaluating the safety and efficacy of ICI consolidation therapy after chemoradiotherapy in China. Our results indicate that ICI consolidation is associated with favorable efficacy and no severe pneumonitis incidence in Chinese patients undergoing both anti‐PD‐1 and anti‐PD‐L1 monoclonal antibody consolidation.

**What this study adds**

Post‐hoc analysis of the Japanese subgroup in the PACIFIC study suggested that consolidation therapy may be associated with increased pneumonitis incidence in Asian patients. However, our findings indicate that consolidation therapy is safe and tolerable in Chinese patients with unresectable stage III NSCLC.

## Graphical abstract

These data indicate that ICI consolidation therapy can achieve favorable short‐term efficacy, while exhibiting good safety and acceptable toxicity profiles in Chinese patients with inoperable stage III NSCLC.

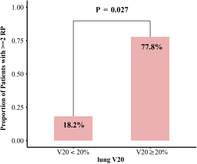

## Introduction

Lung cancer is among the most prominent causes of cancer‐associated death in men and women.^1^ Between 80% and 85% of lung cancer cases are of the non‐small cell lung cancer (NSCLC) subtype, and upwards of 30% of NSCLC patients have locally advanced disease upon initial diagnosis.^2^, ^3^ Historically, the five‐year overall survival (OS) rate for locally advanced NSCLC patients undergoing definitive concurrent chemoradiotherapy (cCRT) treatment has been reported to be 20%–30%.^4^, ^5^, ^6^ Despite countless efforts to improve the prognosis of these patients over recent decades, little progress had been made prior to the advent of immune checkpoint inhibitor (ICI) therapy.

Chemoradiotherapy (CRT) treatment has been shown to drive immunogenic cell death (ICD) in preclinical settings, and this, in turn, has been linked to clinical benefit in the context of combination immunotherapy.^7^, ^8^ In the PACIFIC phase III randomized clinical trial, consolidative programmed death ligand‐1 (PD‐L1) inhibition using durvalumab following cCRT was associated with significant improvements in both the progression‐free survival (PFS) and OS of these NSCLC patients relative to cCRT alone.^9^, ^10^, ^11^ The phase II single‐arm LUN 14–179 study and the phase II DETERRED trial similarly assessed the safety and efficacy of consolidation therapy using pembrolizumab (anti‐PD‐1) or atezolizumab (anti‐PD‐L1) in patients with stage III unresectable NSCLC that did not exhibit disease progression following cCRT.^12^, ^13^ The results of the PACIFIC study have led to the establishment of consolidative ICI therapy as a new standard of care in those with stage III NSCLC.

While ICI consolidation following cCRT is associated with significant survival benefits, it is also linked to relatively high rates of toxicity and adverse events. For example, overall radiation pneumonitis occurred in 33.9% of patients in durvalumab group and 24.8% of patients in placebo group in the PACIFIC study, respectively, with grade 3 and grade 4 disease occurring in 3.4% and 2.6% of these patients, respectively.^10^ Similarly, in the LUN 14–179 study, 17.2% of patients developed grade ≥ 2 pneumonitis, while five (5.4%) developed grade 3–4 pneumonitis, and there was one pneumonitis‐related death.^12^ Pneumonitis also occurred more frequently in Asian patients than in non‐Asian patients in these studies (54.2% vs. 14.1% for any pneumonitis and 5.6% vs. 1.0% for ≥grade 3 pneumonitis, respectively), potentially reflecting ethnicity‐related differences in immune functionality.^14^ Notably, no Chinese cohorts were included in the PACIFIC study, and at present, there is little data available regarding the safety and efficacy of ICI consolidation following CRT in Chinese stage III NSCLC patients. As such, we designed this prospective study in order to evaluate the feasibility, tolerability and efficacy of ICI consolidation after CRT in patients with unresectable stage III NSCLC in China.

## Methods

### Patient eligibility

Safety and efficacy data were prospectively collected for patients with American Joint Committee on Cancer (AJCC) eighth edition stage III NSCLC that had undergone CRT with curative intent at three institutions between September 2018 and January 2020 followed by ICI consolidation therapy without disease progression.

Only patients with histologically/cytologically confirmed unresectable stage III NSCLC and an Eastern Cooperative Oncology Group performance status of 0–2 were eligible for inclusion in the present study. Gene mutation testing was performed for patients with adenocarcinoma carcinoma. This trial protocol was approved by the ethics review boards of Cancer Hospital, Chinese Academy of Medical Sciences and Peking Union Medical College.

### Study design and treatment

Patients in this study underwent cCRT or sequential CRT and chemotherapy using platinum‐based regimens recommended by the NCCN guidelines, including etoposide/cisplatin, paclitaxel/carboplatin, and pemetrexed/cisplatin.

All patients underwent intensity‐modulated radiation therapy (IMRT) or volumetric intensity‐modulated arc therapy (VMAT). The gross tumor volume (GTV) was defined as the extent of primary tumor. GTV of lymph node (GTVnd) was defined as the volume of metastatic lymph nodes in hilar and mediastinum if metastatic lymph nodes were present. The clinical target volume (CTV) included the primary tumor volume plus a 0.6–0.8 cm margin, as well as involved ipsilateral hilum and mediastinal nodal stations. In patients treated via simultaneous integrated boost (SIB) radiotherapy, planning GTV (PGTV) was defined as the sum of GTV and GTVnd with a 5 mm expansion. The prescribed SIB‐IMRT dose was 95% PGTV 60.2 Gy/2.15 Gy/28F, 95% PTV 50.4 Gy/1.8 Gy/28F, and the conventional IMRT dose was 95% PTV 50.0–60 Gy/2–2.2 Gy/23–30F. The mean dose to the lungs (MLD) was not to exceed 18 Gy, and the volume of normal lung receiving >20 Gy (V20) and 30 Gy (V30) was limited to <28% and <20%, respectively.

Patients that underwent ICI consolidation treatment were those without disease progression or radiation pneumonitis (RP) following CRT.

### Evaluation and follow‐up

Baseline patient evaluations consisted of chest and abdominal computed tomography (CT) scans, brain CT or magnetic resonance imaging (MRI) scans, bronchoscopy and radionuclide bone scanning. Positron emission tomography (PET)‐CT scans were also recommended but not mandatory.

Follow‐up evaluation in these patients consisted of patient history, physical examination, chest and abdominal CT conducted every two months during the first year and every 12 weeks thereafter. Additional imaging was performed if recurrence was suspected.

The Response Evaluation Criteria in Solid Tumors (RECIST) version 1.1 criteria were used to assess tumor therapeutic responses.^15^ Treatment‐related toxicities were graded according to the National Cancer Institute Common Toxicity Criteria (NCI CTC), version 5.0. PFS was defined as the period between diagnosis initiation and death, tumor progression, or most recent follow‐up. OS was defined was the period between diagnosis initiation and death, or most recent follow‐up.

### Statistical analysis

Kaplan‐Meier methods were used to assess patient survival. Categorical variables are given as numbers and percentages, while continuous variables are given as means ± standard deviations. Data were compared via chi‐squared tests and Student's *t‐*tests, as appropriate. All *P*‐values were two‐sided, with *P* < 0.05 as the significance threshold. STATA v16.0 (StataCorp, TX, USA) was used for all statistical testing in this study.

## Results

### Cohort characteristics

In total, 20 NSCLC patients were enrolled in this study between September 2018 and January 2020. These patients had a median age at diagnosis of 61 years (range: 43–78). Of these patients, 16 (80.0%) were male, and 14 (70.0%) had a history of smoking. One patient (5.0%) had stage IIB disease, seven patients (35.0%) had stage IIIA disease, eight patients (40.0%) had stage IIIB disease, and four patients (20.0%) had stage IIIC disease. The most common primary diagnoses in these patients were squamous cell carcinoma (45.0%) and adenocarcinoma (50.0%). No *EGFR* or *ALK* mutation were detected in patients with adenocarcinoma. The characteristics of patients are shown Table 1.

**Table 1:** Baseline participant characteristics in the overall patient cohort

N (%)	Overall (*n* = 20)
Age (median,range)	61 (43–78)
Gender	
Female	4 (20.0)
Male	16 (80.0)
Smoking	
Yes	14 (70.0)
No	6 (30.0)
Histology	
ACC	9 (45.0)
SCC	10 (50.0)
NSCLC	1 (5.0)
Stage	
IIB	1 (5.0)
IIIA	7 (35.0)
IIIB	8 (40.0)
IIIC	5 (20.0)
SIB	
Yes	15 (75.0)
No	6 (25.0)
cCRT	
Yes	17 (85.0)
No	3 (15.0)
Does (Gy, median, range)	60.2 (50.0–65.0)
MLD (Gy, median, range)	11.40 (1.49–15.00)
Lung V20%	19.7 (1.76–27.0)
Lung V30%	14.8 (1.37–20.3)

ACC, adenocarcinoma; cCRT, concurrent chemoradiotherapy; Gy, Gray; MLD, mean dose to the lungs; NSCLC, non‐small cell lung cancer; SCC, squamous cell carcinoma.

### Treatment

Of the patients enrolled in this study, 17 (85.0%) received cCRT and three (15.0%) received sequential CRT. The cCRT regimen included cisplatin and etoposide (*n* = 10, 50.0%), pemetrexed and cisplatin (*n* = 6, 30.0%), or paclitaxel and nedaplatin (*n* = 1, 5.0%). The median radiation dose was 60.2 Gy (range: 50 Gy–60.2 Gy). In total, 15 patients received SIB‐IMRT and five received conventional IMRT. The median MLD was 11.40 Gy (range: 1.49 Gy–15.00 Gy), the median lung V20 was 19.7% (range: 1.76%–27.0%) and V30 was 14.8% (range: 1.37%–20.3%).

The median time between radiotherapy termination and consolidation therapy initiation was 40.5 days (range: 1–85 days). A total of 10 patients (50.0%) were treated via PD‐L1 monoclonal antibody therapy (durvalumab, 10 mg/kg biweekly), while 10 patients (52.4%) were treated via PD‐1 monoclonal antibody therapy (terepril, *n* = 5, 240 mg triweekly; sintilizumab, *n* = 3, 200 mg triweekly; nivolumab, *n* = 1, 240 mg biweekly; pembrolizumab, *n* = 1, 200 mg triweekly).

### Safety

All adverse events in these patients are reported in Table 2. Adverse events of grade 3 or higher were rare during CRT, with only six patients (30.0%) experiencing grade 3 leukopenia, and with no other reports of radiation‐associated grade 3 adverse events. The adverse events of patients are shown in Table 2.

**Table 2:** All‐cause adverse events in study patients

Grade of adverse event, No. (%)	All grade	Grades 1	Grades 2	Grades 3
Phase of chemoradiation				
Esophagitis	13 (75.0)	9 (45.0)	4 (20.0)	0
Anemia	7 (35.0)	4 (20.0)	3 (15.0)	0
Thrombocytopenia	6 (40)	7 (35.0)	2 (10.0)	0
Leukopenia	17 (81.0)	3 (15.0)	7 (35.0)	6 (30.0)
Radiation dermatitis	6 (30)	5 (25.0)	1 (5.0)	0
Phase of consolidation ICI				
Pneumonitis	16 (80.0)	7 (35.0)	9 (45.0)	0
Fatigue	2 (10.0.)	2 (10.0)	0	0
Cough	2 (10.0)	2 (10.0)	0	0
Fever	2 (10.0)	2 (10.0)	0	0
Thyroid dysfunction	1 (5.0)	1 (5.0)	0	0
Chest pain	1 (5.0)	0	1 (5.0)	0

**Figure 1:**
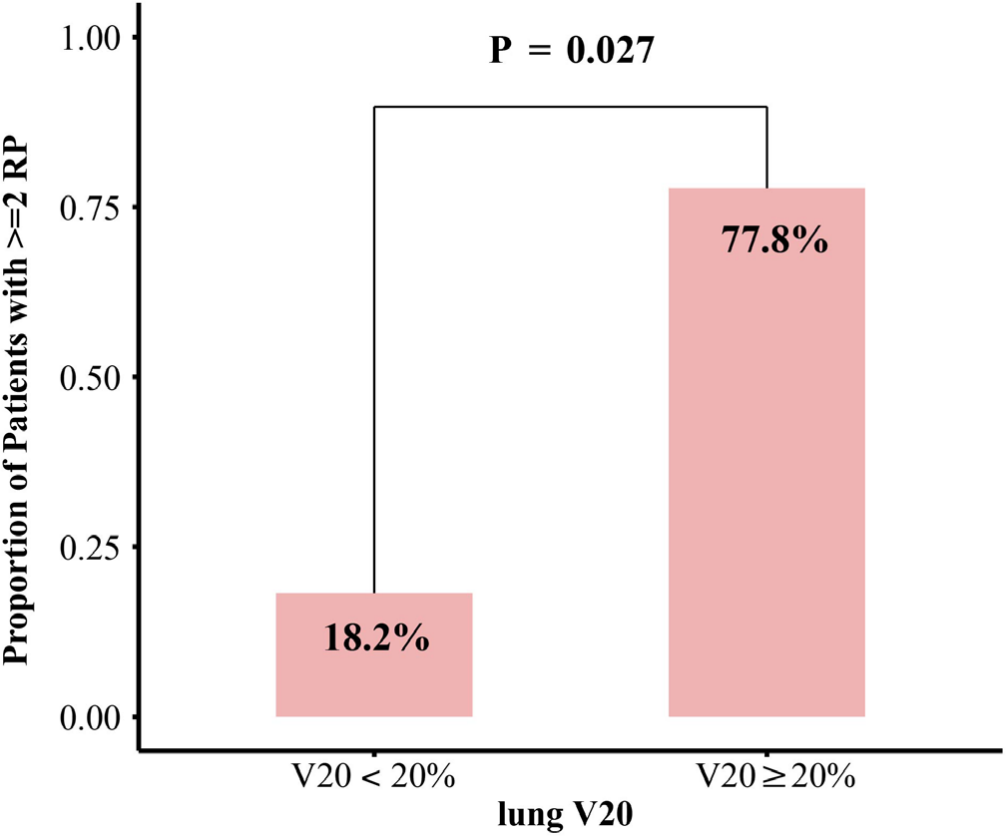
The relationship between dose parameters (V20) and radiation pneumonitis.

During ICI consolidation therapy, 80.0% (*n* = 16) of patients had pneumonitis of grade 1 or higher, of whom seven (35.0%) had grade 1 pneumonitis and nine (45.0%) had grade 2 pneumonitis. The median time to pneumonitis onsite following ICI initiation was 72 (range: 23–120) days. Lung V20 ≥ 20% was associated with higher grade 2 pneumonitis (77.8% [in ≥20% group] vs. 18.2% [in <20% group], *P* = 0.027, Fig 1). All patients who experienced grade 2 pneumonitis were treated with prednisone (1 mg/kg) for >1 month.

Seven patients experienced additional immune‐related toxicities, including six grade 1 toxicities and one grade 2 toxicity (chest pain). No grade 3 or higher immune‐related adverse events occurred in this study.

### Response and survival

**Figure 2:**
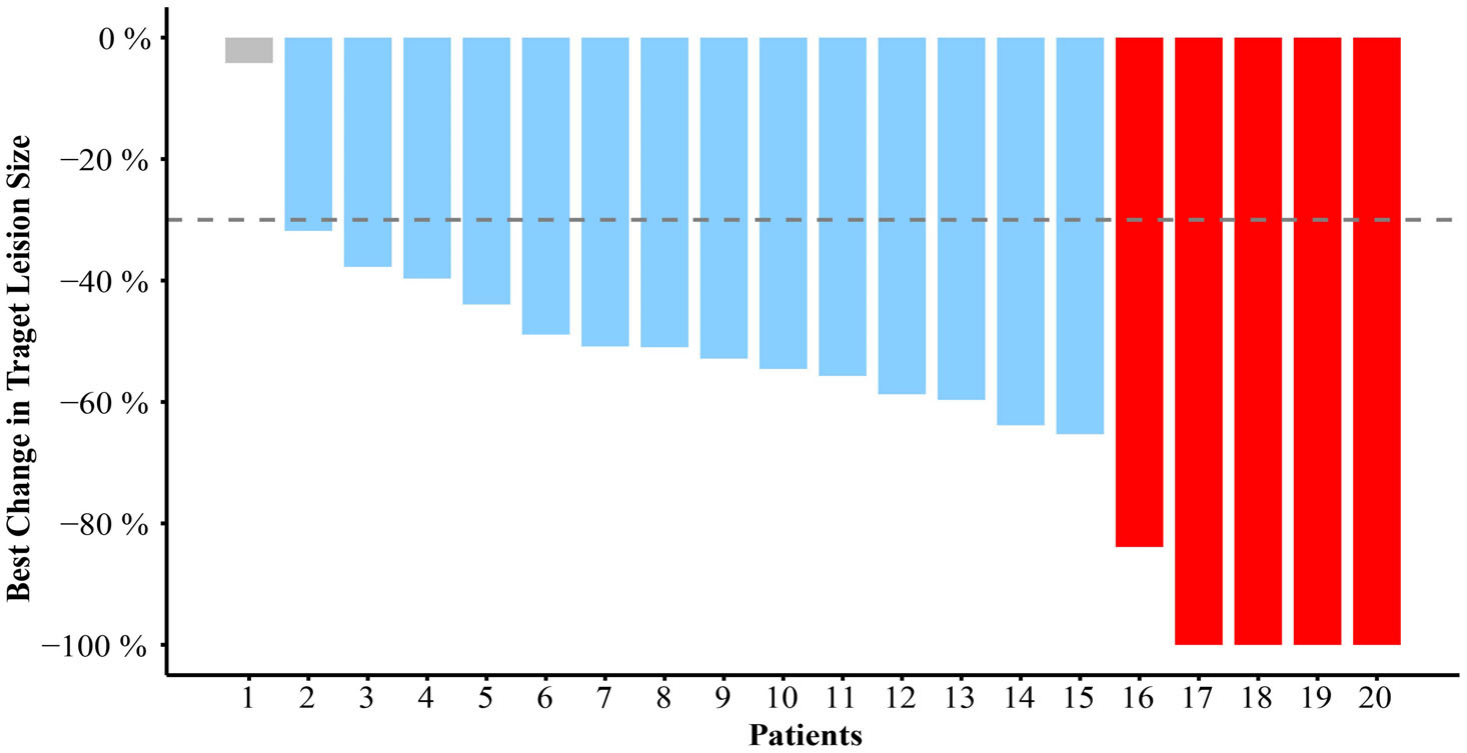
The best responses of patients for ICI consolidation after CRT. The dashed line corresponds to a 30% reduction in target lesion size as defined by the RECIST 1.1 criteria. 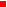, CR; 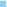, PR; 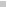, SD.

The median follow‐up period in this study was 11.3 (range: 6.2–21.8) months. A partial response (PR) was achieved in 14 (66.7%) patients, while complete response (CR) was achieved in five (25.0%) patients for an ORR (CR + PR) of 95.0% (Fig 2, two cases with CR are shown in Figs S1 and S2). Three patients ultimately developed metastatic disease within the study period, exhibiting metastases in the lung and bone and one patient died. The 12‐month PFS of these patients was 89.5% (95% CI: 76.7%–100%), and 12‐month OS was 100%.

## Discussion

Immunotherapy has revolutionized cancer treatment, with ICIs including monoclonal anti‐PD‐1, anti‐PD‐L1, and anti‐CTLA‐4 having significantly improved survival outcomes in lung cancer and melanoma patients.^16^, ^17^, ^18^ In patients with advanced forms of lung cancer patients, combination ICIs and chemotherapy can significantly improve therapeutic efficacy and patient survival. Keynote‐189 and Keynote‐407 have revealed that the combination of pembrolizumab with chemotherapy significantly improved advanced NSCLC patient survival.^18^, ^19^ The combination of ICIs with concurrent or sequential CRT has been definitively established as an optimal treatment strategy for patients with locally advanced NSCLC, and this approach is generally well tolerated. Herein, we found that 12‐month PFS in patients undergoing ICI consolidation therapy following CRT was 89.5%, which was promising given that the 12‐month PFS observed in the PACIFIC study was 55.7%.^10^

The PACIFIC trial was a randomized, placebo‐controlled, double‐blinded, multicenter analysis of 713 locally advanced NSCLC patients which found that durvalumab consolidation significantly enhanced PFS following CRT regardless of PD‐L1 expression status (median PFS: 16.8 months vs. 5.6 months; hazard ratio [HR] = 0.52, 95% CI: 0.42–0.65, *P* < 0.001). The LUN 14‐179 study was a phase II single‐arm prospective study evaluating pembrolizumab consolidation therapy safety and efficacy in stage III unresectable NSCLC patients not exhibiting disease progression after cCRT within a 12‐month period. At a median follow‐up of 18.6 months, median PFS was 17.0 months (95% CI: 11.9–NX), with 12–18‐ and 24‐month PFS rates of 60.2%, 49.9%, and 44.6%, respectively. At the time of this analysis, median OS had not yet been reached, and 12–18‐ and 24‐month OS rates were 81.0%, 68.0%, and 61.9%, respectively.^20^ These two trials confirmed that ICIs including both anti‐PD‐1 and anti‐PD‐L1 could be used as consolidation therapy to prolong OS and PFS of patients with locally advanced NSCLC. In this study, we included patients treated with both anti‐PD‐1 and anti‐PD‐L1. A prior meta‐analysis of 112 trials and 19 217 patients observed comparable fatality rates for both of these antibodies at 0.36% (anti‐PD‐1) and 0.38% (anti‐PD‐L1).^21^ We found similar rates of grade 2 pneumonitis in patients treated with anti‐PD‐1 and anti‐PD‐L1(40.0% vs. 50.0%, *P* = 1.000). We observed a 95.2% ORR (CR + PR) in this study. In prior studies including some phase III trials, traditional CRT was associated with ORRs ranging from 35% to 70%, and CR rates were generally <10% (Table 3). Consolidation ICI therapy is thus a promising therapeutic approach in Chinese NSCLC patients.

**Table 3:** Studies of chemoradiotherapy for the treatment of stage III NSCLC

Study	Population	Radiation dose (Gy)	CR (%)	PR (%)	ORR (%)	Median PFS, (months)	Median OS (months)
RTOG 9410^28^	II/III NSCLC (*n* = 610)	63	42	28	70	NR	17.0
CALGB 39801^29^	III NSCLC (*n* = 182)	66	7	59	67	NR	12.0
LUN 01–24^30^	III NSCLC (*n* = 74)	59.4	NR	NR	NR	NR	23.2
PROCLAIM^31^	IIIA/B NSCLC (*n* = 598)	60–66	1.3	34.6	35.9	11.4	26.8
RTOG 0617^32^	IIIA/B NSCLC (*n* = 228)	60	NR	NR	NR	11.8	28.7
KCSG‐LU05‐04^30^	IIIA/B NSCLC (*n* = 437)	66	2.9	40.2	43.1	9.1	21.8

mo, months.

Limited data is available regarding immunotherapy toxicity following radical CRT treatment in stage III NSCLC patients. In the LUN 14‐179 phase II trial, 17.2% of patients experienced pneumonitis of grade ≥2, with 10.8%, 4.3%, and 1.1% of patients suffering from grade 2, 3, and 4/5 pneumonitis, respectively.^12^ In the PACIFIC study, 33.9% and 24.8% of patients experienced any form of pneumonitis and RP, respectively, with 3.4% and 2.6% of patients experiencing grades 3 and 4 pneumonitis, respectively.^10^ While overall pneumonitis incidence in our study was markedly higher (73.3%), we found that only 53.3% of patients experienced symptomatic pneumonitis (grade > 2), and no patients experienced severe pneumonitis (grade > 3). This is in line with the results of the post‐hoc analyses of the Japanese subgroup in the PACIFIC trial, in which 47.9% of patients experienced pneumonitis.^22^ These results thus suggest that ICI consolidation therapy is safe and feasible in Chinese patients. A Japanese retrospective analysis of 108 patients found that rates of grade 1, 2, and 3 pneumonitis were 58%, 26%, and 1% with a V20 constraint of 35%.^23^ Another analysis of 31 patients found that pneumonitis occurred in 17.2% of all patients, and was of at least grade 3 in 6.9% of these patients.^24^ In Table 4 we have summarized the incidence of pneumonitis associated with ICI consolidation therapy in Asian patients in different studies. These analyses indicate that the pneumonitis rates range from 17.2% to 85% (pooled rate: 67.1%), with grade 3 or higher pneumonitis occurring in 0%–14.3% of patients (pooled rate: 4.4%, Table 4). The increase in pneumonitis rates in Asian populations relative to non‐Asian populations may suggest that there are differences in the immune responses in these two groups. Finally, the PACIFIC study specified a lung V20 of ≤35% or a mean lung dose of ≤20 Gy. In the real‐world of Korea study, researchers found that V20 ≥ 35% and MLD ≥ 20 Gy were associated with more grade 2 or higher‐grade RP (61.9% vs. 15.0%; 70.6% vs. 15.9%).^25^ In our study, the higher V20 was associated with more grade 2 RP (V20 ≥ 20% vs. V20 < 20%; 77.8% vs. 18.2%, *P* = 0.027). We therefore propose that the total lung V20 to highest thresholds such as 28% or less is reasonable.

**Table 4:** Pneumonitis in Asian stage III NSCLC following ICI consolidation therapy

Study	Country (region)	Population	All pneumonitis (%)	G2 pneumonitis (%)	G3–5 pneumonitis (%)
Fukui *et al*.^23^	Japan	108	85	26.0	2.0
Chu *et al*.^24^	China (Taiwan)	31	17.2	NR	6.9
Jung *et al*.^25^	Korea	21	81	42.9	14.3
Sakaguchi *et al*.^33^	Japan	73	73.9	NR	5.5
PACIFIC Japan cohort^14^	Japan	112	54.2	NR	5.6
Current study	China	20	80.0	45.0	0
Pooled data	Asian	365	67.1	31.2	4.4
PACIFIC	Multicountry	476/709	33.9	NR	3.4
LUN14‐179	USA	92	NR	10.8	6.5

G2, grade 2; G3–5, grades 3–5; NR, not reported.

Further research regarding optimal ICI timing relative to radiotherapy administration is required. Updated OS data from the PACIFIC trial indicate that patients administered durvalumab ≤14 days following radiation survived for longer than patients treated within ≥14 days (HR 0.43, 95% CI: 0.26–0.66 vs. 0.79, 95% CI: 0.61–1.02).^11^ This suggests that earlier ICI administration is probably linked to maximal survival benefit. In a different phase I prospective multicenter nonrandomized controlled trial, pembrolizumab was combined with cCRT (weekly carboplatin and paclitaxel with standard radiotherapy). In this study, patients receiving at least one dose of pembrolizumab (*n* = 21) had a median PFS of 18.7 months (95% CI: 11.8–29.4), and the six‐ and 12‐month PFS of this patient group was 81.0% (95% CI: 64.1%–97.7%) and 69.7% (95% CI: 49.3%–90.2%), respectively. The median PFS for patients receiving two or more doses of pembrolizumab (*n* = 19) was 21.0 (95% CI: 15.3–infinity) months.^26^ At present, the PACIFIC 2 trial (NCT03519971, phase III study) is being conducted in order to assess the efficacy and safety of durvalumab combined with concurrent platinum‐based chemoradiation in patients with unresectable stage III NSCLC.

Herein, we explored the use of ICI consolidation therapy for the treatment of Chinese patients with inoperable stage III NSCLC. However, our sample size was limited, with just 20 patients having been included in this analysis. Of these patients, only 17 underwent PACIFIC model treatment, with the remaining three undergoing ICI consolidation following sequential chemoradiotherapy. Many patients in China with inoperable stage III NSCLC elect to undergo sequential radiotherapy and chemotherapy, while certain patients elect to undergo radiotherapy alone due to advanced age or poor overall health. Further research regarding ICI consolidation after sequential CRT or radiotherapy is still required. Furthermore, the majority of patients in the present study did not undergo analyses of intratumoral PD‐L1 expression. Results from the PACIFIC study suggest that patients with >1% PD‐L1 expression were more likely to benefit from immunotherapy.^27^ As such, PD‐L1 expression in these patients should be evaluated further in subsequent studies.

In conclusion, ICI consolidation therapy is associated with favorable short‐term efficacy, good safety, and acceptable toxicity profiles in Chinese patients with unresectable stage III NSCLC. While these patients experienced a higher incidence of pneumonitis than other previously reported populations, no severe pneumonitis was reported. Overall, our findings are consistent with prior studies of Asian populations and those of other ethnicities.

## Disclosure

All authors have no conflict of interest to disclose.

## Supporting information


**Supplementary Figure S1** This patient (Case 1) presented with T1cN3M0 adenocarcinoma and received Terepril consolidation after cCRT. This patient achieved complete response for both primary and lymph node target lesions.

**Supplementary Figure S2** This patient (Case 2) presented with T4N3M0 adenocarcinoma and received Durvalumab consolidation following concurrent chemoradiation and induction chemotherapy. This patient achieved complete response for both primary and lymph node target lesions.
