# Supplementary figures and images for: Efficacy and safety of immune checkpoint inhibitor consolidation after chemoradiation in patients of Asian ethnicity with unresectable stage III non‐small cell lung cancer: Chinese multicenter report and literature review
Source: Thorac Cancer. 2020 Aug 24;11(10):2916–23. doi: 10.1111/1759-7714.13631 (PMC7529561; doi:10.1111/1759-7714.13631)

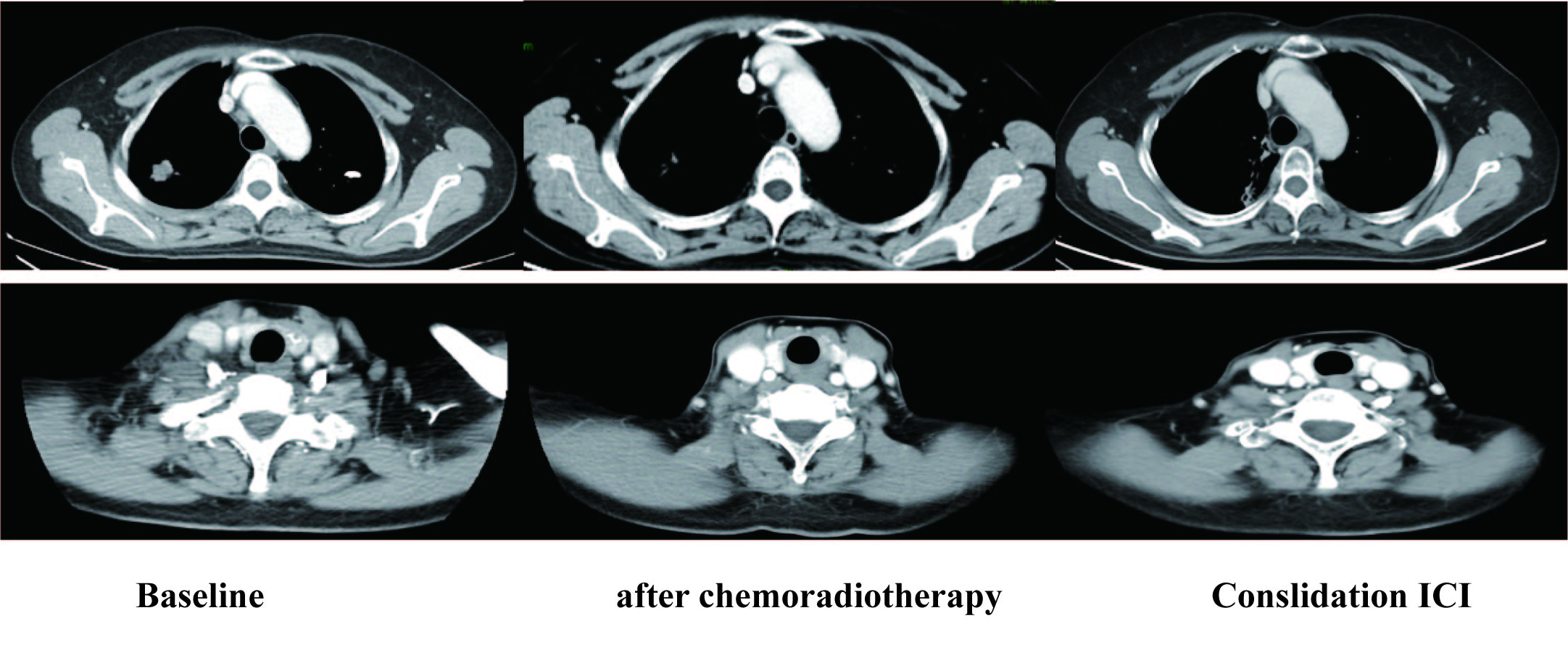

Supplement: Supplementary file 1 — Supplementary Figure S1 This patient (Case 1) presented with T1cN3M0 adenocarcinoma and received Terepril consolidation after cCRT. This patient achieved complete response for both primary and lymph node target lesions. [file TCA-11-2916-s001.jpg]

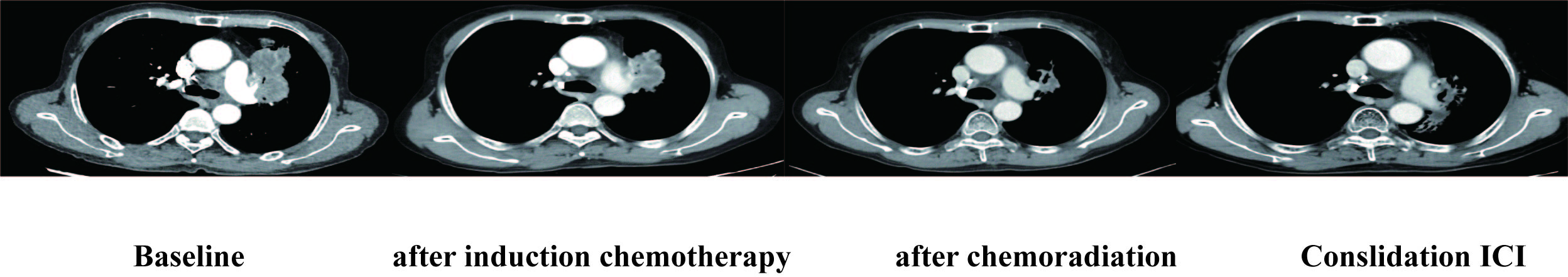

Supplement: Supplementary file 2 — Supplementary Figure S2 This patient (Case 2) presented with T4N3M0 adenocarcinoma and received Durvalumab consolidation following concurrent chemoradiation and induction chemotherapy. This patient achieved complete response for both primary and lymph node target lesions. [file TCA-11-2916-s002.jpg]
